# The role of epigenetics in pulmonary fibrosis: recent advances in mechanistic insights and therapeutic implications

**DOI:** 10.3389/fmolb.2025.1647300

**Published:** 2025-07-17

**Authors:** Jingru Huang, Jianfeng Qin, Yuguang Zhu, Ao Shen

**Affiliations:** Guangzhou Municipal and Guangdong Provincial Key Laboratory of Molecular Target and Clinical Pharmacology, NMPA and State Key Laboratory of Respiratory Disease, School of Pharmaceutical Sciences and the Fifth Affiliated Hospital, Guangzhou Medical University, Guangzhou, Guangdong, China

**Keywords:** idiopathic pulmonary fibrosis, epigenetic regulation, DNA methylation, RNA methylation, histone modifications, non-coding RNA

## Abstract

Pulmonary fibrosis (PF) is a fatal disease characterized by progressive fibrosis of lung tissue, with a key pathological feature of excessive accumulation of extracellular matrix. PF occurs from complicated origins, while emerging findings have suggested the involvement of the environmental factors in the risk of PF through epigenetic regulation. This article will discuss how recent advances in epigenetic alterations of DNA methylation, RNA methylation, histone modifications, and non-coding RNAs contribute to PF development through molecular mechanisms and cellular processes, including fibroblast-to-myofibroblast transition (FMT), epithelial-to-mesenchymal transition (EMT), alveolar epithelial cell injury and immune cell interactions in the past 5 years.

## 1 Introduction

Pulmonary fibrosis (PF) is a form of interstitial lung disease (ILD) that causes scarring in the lungs (Richeldi et al., 2017; Raghu et al., 2022). PF becomes a global health threat with very limited therapeutic options available, as abnormal extracellular matrix (ECM) deposition and damage to the alveolar structure will ultimately lead to respiratory failure (Richeldi et al., 2017; Raghu et al., 2022). PF can arise from a variety of causes, including both identifiable factors and idiopathic origins (Koudstaal et al., 2023; Kondoh and Inoue, 2025). Accordingly, PF can be classified into various types based on its etiology. The most common form is idiopathic pulmonary fibrosis (IPF), which has unknown causes. In addition to IPF, secondary PF can result from chronic viral infections or autoimmune diseases, such as rheumatoid arthritis and systemic sclerosis. Environmental exposures also play a critical role in some cases of PF. Factors such as radiation damage, certain types of drugs (e.g., amiodarone, bleomycin), and long-term inhalation of hazardous materials are all involved in PF development. Examples include occupational exposures to asbestos or silica, as well as environmental stressors such as cigarette smoking or particulate matte with diameters of 2.5 μm and smaller (PM2.5) (Koudstaal et al., 2023; Kondoh and Inoue, 2025).

Animal models have been widely used to aid in understanding those diverse PF etiologies, ranging from idiopathic to environmental or genetic forms (O’Dwyer and Moore, 2018; Yanagihara et al., 2020). The bleomycin-induced model recapitulates key features of IPF, though it lacks complete usual interstitial pneumonia patterns. Models using silica, fluorescein isothiocyanate (FITC), radiation, or viral-based transgenes simulate occupational silicosis-related fibrosis, radiation pneumonitis, and genetic PF (e.g., Sftpc mutations encoding surfactant protein C), respectively. Genetically modified mice, such as knockouts of telomerase reverse transcriptase (TERT) mimic familial PF, while repeated bleomycin dosing better reflects progressive fibrosis (Li S. et al., 2022).

Complementing animal models, cellular models offer mechanistic insights (Yanagihara et al., 2020; Li et al., 2024; Kolanko et al., 2024). Commonly used *in vitro* cell models for studying PF include various types of lung cells. Human or animal lung fibroblasts (e.g., MRC-5) are frequently used to investigate fibroblast differentiation into myofibroblasts and the impact of tissue stiffness. Alveolar epithelial cells, such as primary AT2 cells or the A549 cell line, are also employed to study epithelial-mesenchymal transition (EMT) and barrier properties. TGF-β1 and bleomycin treatments represent two most common cellular models to study ECM deposition and inflammation in IPF and drug-induced PF. Additionally, co-culture models that combine fibroblasts and epithelial cells, or macrophages (e.g., RAW264.7), allow for the study of cell-cell interactions. These models help researchers understand the underlying mechanisms of PF and test potential therapies.

Epigenetic regulation, including but not limited to DNA methylation, RNA modifications, histone modifications and non-coding RNA (ncRNA), is emerging to be key regulators of lung cell responses to aforementioned external stresses, linking environmental exposures with fibrotic phenotypes. The epigenetic frameworks and disease mechanisms across major publications are concisely summarized in previous reviews (Koudstaal et al., 2023; Moss et al., 2022; Wang et al., 2023), so this review will concentrate on the most updated mechanistic aspects of the last 5 years (2019–2025) in regard to current epigenetic regulatory mechanisms for PF pathology, therapeutic implications and future research directions.

## 2 DNA methylation

**FIGURE 1:**
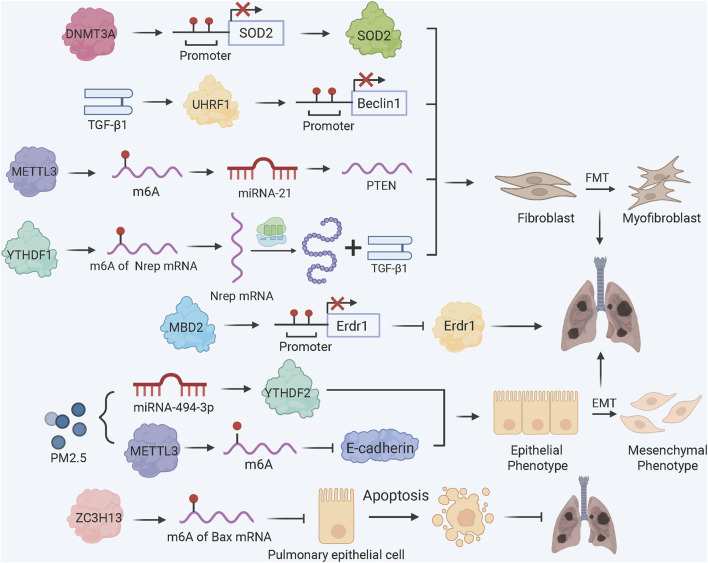
DNA and RNA Methylation Dynamics in PF Pathogenesis. TGF-β1 induces UHRF1 upregulation, promoting methylation of Beclin 1 promoter to inhibit autophagy and enhance FMT; DNA methylation reader MBD2 binds the methylated Erdr1 promoter to suppress its expression, amplifying TGF-β/Smad signaling and FMT. METTL3-mediated m6A modification drives MSC differentiation into myofibroblasts via the miRNA-21/PTEN pathway; YTHDF1 bind to m6A-modified Nrep mRNA enhances TGF-β1 secretion to accelerate FMT; PM2.5 exposure reduces miRNA-494-3p expression, allowing YTHDF2 to bind m6A-modified CDH1 mRNA and induce EMT; ZC3H13 promotes m6A methylation of Bax mRNA, reducing epithelial apoptosis via YTHDC1-mediated destabilization.

DNA methylation refers to the covalent attachment of a methyl group to DNA bases, generally on the fifth position of cytosine (5 mC), mainly restricted to CpG sites in mammalian. DNA methylation is functionally dynamic and balanced by DNA methyltransferases (DNMTs) and demethylases to influence gene expression through chromatin accessibility (Sergeeva et al., 2023; Loaeza-Loaeza et al., 2020). The mammalian DNMTs mainly includes DNMT1, DNMT3A and DNMT3B, among which DNMT3A and DNMT3B function as *de novo* methylation enzymes while DNMT1 maintains DNA methylation signatures during DNA replication. DNA methylation can be removed via two main mechanisms. Passive demethylation occurs when methylation patterns is disrupted during DNA replication, leading to the dilution of methylated cytosines. Active demethylation involves the oxidation of 5 mC by the Ten-eleven translocation (TET) family of enzymes (TET1, TET2, and TET3), then oxidized cytosines are excised by thymine DNA glycosylase (TDG), and the original cytosine is restored through base excision repair. DNA methylation modification in PF plays a pivotal role in disease pathogenesis (Figure 1), particularly through fibroblast-myofibroblast transformation (FMT) (Duan et al., 2022; Rajasekar et al., 2021).

Fibroblast activation in PF is marked by global DNA hypermethylation and upregulation of the methylation reader protein MBD2 (methyl-CpG-binding domain 2) (Wang et al., 2022). TGF-β1 induces a positive feedback loop where TβRI/Smad3 signaling drives MBD2 upregulation, leading to hypermethylation of the Erdr1 (erythroid differentiation regulator 1) promoter. MBD2-bound methylated Erdr1 suppresses its anti-fibrotic function, enhancing TGF-β/Smad signaling and FMT (Wang et al., 2022). Concurrently, TGF-β1 upregulates UHRF1 (ubiquitin-like PHD and RING finger domain 1), which promotes Beclin 1 promoter methylation to inhibit autophagy and accelerate FMT *in vitro* and *in vivo* (Cheng et al., 2022).

Clinically, fibroblasts from IPF patients are hypermethylated at the CDKN2B gene locus, which functions as a cyclin dependent kinase inhibitor, and therefore its reduced expression resulted in myofibroblast differentiation via SRF (serum response factor) and MRTF-A (myocardin-related transcription factor A) but not by cell proliferation (Scruggs et al., 2018). Conversely, FOXL1 (forkhead box L1), which is a lung fibroblast specific transcription factor, keeps low DNA methylation status at its promoter via super enhancer formation in quiescent fibroblasts. Upregulation of FOXL1 in IPF patients is correlated by increased expression of TAZ/YAP and PDGFRα, enhancing fibroblast migration and collagen deposition (Miyashita et al., 2020). Recently, it has been reported that DNMT3A drives PF by repressing SOD2 (superoxide dismutase 2), a critical antioxidant enzyme. This was achieved by directly binding of DNMT3A to the SOD2 promoter, inducing hypermethylation and SOD2 silencing, which exacerbated oxidative stress and fibroblast proliferation (Wang et al., 2025).

## 3 RNA methylation

RNA methylation is a regulatory mechanism at the post transcriptional level. Currently, over 150 RNA modifications have been identified (Zhang et al., 2022b). RNA methylation, especially N6-methyladenosine (m6A) as the most abundant modification in mammalian mRNA, coordinates PF by influencing mRNA stability, translation, and ncRNA activity. The m6A “writer” complex contains three core proteins: methyltransferase like 3 (METTL3), METTL14, and Wilms tumor associated protein 1 (WTAP). The “Erasers” include FTO and ALKBH5, which reversibly mediate demethylation. Currently, three types of “readers” have been identified: the YTH-RNA binding domain family, including YTHDF1-3 and two YTH domain containing proteins (YTHDC1-C2) (Zhou et al., 2020; Jiang et al., 2021).

m6A methylation has been demonstrated to be involved in fibrosis in different cellular and animal models (Figure 1) (Ilieva and Uchida, 2022). METTL3, the principal m6A methyltransferase, plays the central role in m6A-dependent PF progression. For instance, METTL3 is critical for FMT. In bleomycin (BLM)-induced PF models, METTL3 knockdown decreases overall m6A levels, thereby hindering the differentiation of myofibroblasts and the deposition of collagen both *in vitro* and *in vivo* (Zhang et al., 2021). In mesenchymal stem cells (MSCs) residing in the lung, TGF-β1 increases METTL3 expression, resulting in m6A modification of PTEN mRNA. This promotes miRNA-21-mediated PTEN degradation, activating AKT signaling and MSCs differentiation into myofibroblasts (Lu et al., 2023). In arsenic-associated PF, YTHDF1 recognizes m6A sites on Nrep (neuronal regeneration-related protein) mRNA, enhancing its translation and TGF-β1 secretion to accelerate FMT (Wang et al., 2024).

For epithelial cell fate, the m6A methyltransferase ZC3H13 protects against PF by promoting m6A methylation of Bax mRNA, which is recognized by YTHDC1 to destabilize Bax protein and inhibit alveolar epithelial cell apoptosis (Guan et al., 2024). In contrast, exposure to PM2.5 triggers downregulation of miRNA-494-3p as well as METTL3-dependent m6A modification of CDH1 mRNA, which YTHDF2 (a target gene of miRNA-494-3p) binds to, resulting in the degradation of E-cadherin and the transition from epithelial to mesenchymal states (Ning et al., 2022). In silicosis PF models, global m6A levels are elevated, with differential methylation of 359 genes (e.g., phagosome and apoptosis pathways), highlighting m6A’s role in inflammatory fibrosis (Zhang et al., 2022a).

## 4 Histone modifications

Histones, as the core component of nucleosomes, play an important role in gene expression regulation through post-translational modifications. Common types of histone modifications include methylation, acetylation, phosphorylation, and ubiquitination, which are dynamically regulated through the catalysis of specific enzymes such as histone acetyltransferases (HATs), histone deacetylases (HDACs), and histone lysine methyltransferases (KMTs) (He et al., 2021). Histone methylation and acetylation, rewire gene expression networks by altering nucleosome dynamics, thereby influencing FMT and EMT in PF (Velagacherla et al., 2022; Korfei et al., 2022).

**FIGURE 2:**
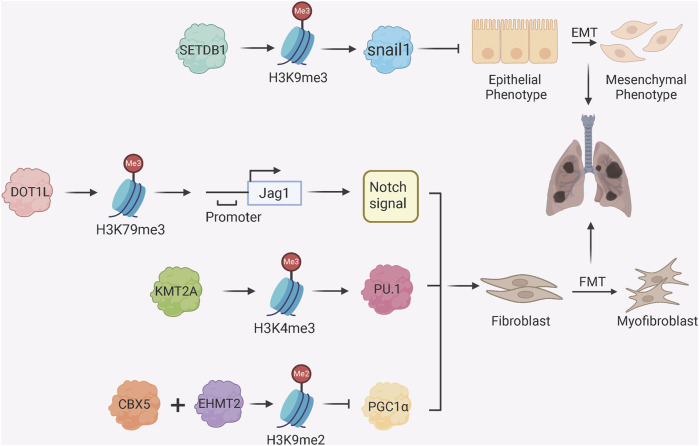
Histone Methylation Regulators in Fibroblast Activation and EMT. SETDB1 catalyzes H3K9me3 at the Snai1 promoter to repress EMT; DOT1L mediates H3K79me3 enrichment at the Jag1 promoter, activating Notch signaling and fibrotic responses; KMT2A catalyzes H3K4me3 at the PU.1 promoter, activating PU.1 and FMT; CBX5 and EHMT2 (G9a) induce H3K9me2 at the PGC1α promoter, promoting FMT by suppressing mitochondrial function.

Histone methylation has a dual role in PF pathogenesis (Figure 2). The histone methyltransferase SETDB1 inhibits EMT through catalysis of histone H3 lysine 9 trimethylation (H3K9me3) at the Snai1 promoter, a key transcriptional factor which triggers EMT initiation, to inhibit Snai1 transcription and maintain epithelial identity (Liu et al., 2022). The histone methyltransferase DOT1L induces H3K79me3 at the Jag1 promoter to upregulate Notch ligand Jagged1 and hyperactivate Notch signalling to promote fibroblast activation and collagen deposition in a BLM-induced PF model (Yang D. et al., 2022). Similarly, the histone methyltransferase KMT2A regulates transcription factor PU.1 transcription in fibroblasts through H3K4me3 at promoter to regulate fibroblast activation (Lyu et al., 2025). Furthermore, the histone methyltransferase EHMT2 (commonly known as G9a) works with the chromobox homolog 5 (CBX5), a gene silencer that binds to H3K9me to establish H3K9me2 modification at the PGC1α promoter and inhibit mitochondrial biogenesis and activate myofibroblasts through YAP/TAZ-mediated pathway (Ligresti et al., 2019). Silencing of G9a in fibroblasts attenuates collagen accumulation after injury, and an inhibitor of G9 elevates PGC1α expression limiting fibrosis progression (Ligresti et al., 2019). Consistently, silencing CBX5 in fibroblasts reduced the development of bleomycin-induced lung fibrosis and genes associated with fibroblast fibrotic activation (Hong et al., 2025).

**FIGURE 3:**
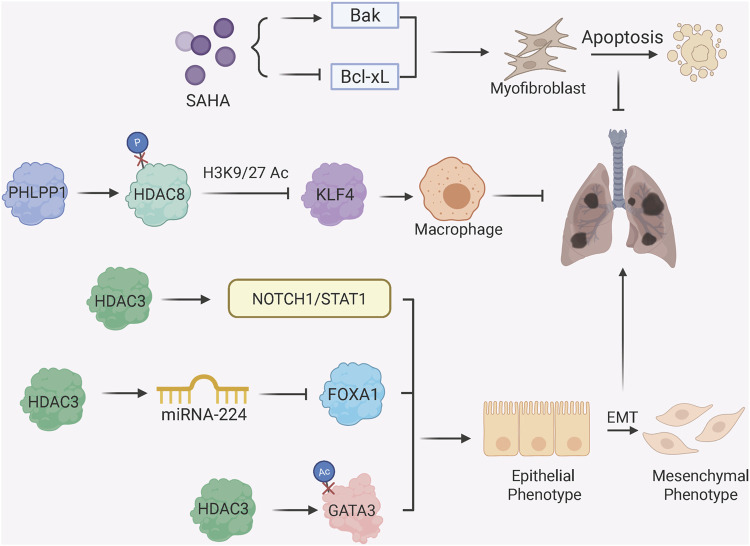
Histone Deacetylation and Fibrotic Phenotypes. HDAC inhibitor SAHA induces myofibroblast apoptosis by upregulating Bak and downregulating Bcl-xL; protein phosphatase PHLPP1 dephosphorylates HDAC8 to enhance deacetylase activity, thereby suppress KLF4 expression in alveolar macrophages which otherwise promotes FMT; HDAC3 promotes EMT through multiple mechanisms: activating Notch1/STAT1 signaling via deacetylation of NICD1 and STAT1; regulating miRNA-224/FOXA1 axis to enhance mesenchymal gene expression; stabilizing GATA3 via deacetylation to sustain EMT.

Histone deacetylation, mediated by HDACs, regulates fibrosis by condensing chromatin and repressing anti-fibrotic or pro-fibrotic genes (Figure 3) (Korfei et al., 2022). Earlier study shows that HDAC inhibitor SAHA (suberoylanilide hydroxamic acid) induces myofibroblast apoptosis in IPF by upregulating pro-apoptotic Bak and downregulating Bcl-xL, with associated changes in histone acetylation at their regulatory regions (Sanders et al., 2014). HDAC3, a key pro-fibrotic enzyme, promotes EMT through multiple mechanisms: it deacetylates and stabilizes Notch intracellular domain 1 (NICD1) and STAT1 to activate Notch1/STAT1 signaling (Zheng et al., 2022), suppresses miRNA-224 to upregulate mesenchymal transcription factor FOXA1 (forkhead box A1) (Jeong et al., 2022), and prevents GATA3 degradation via deacetylation, sustaining EMT in alveolar epithelial cells (Xiong et al., 2023). On the other hand, PHLPP1 (pleckstrin homology domain and leucine-rich repeat protein phosphatase 1) has recently been identified as a protective factor against PF. In alveolar macrophages, PHLPP1 directly binds to and dephosphorylates HDAC8 at serine 39, thereby enhancing HDAC8 activity. This subsequently suppresses the expression of Kruppel-like factor 4 (KLF4) through decreased histone acetylation, which in turn inhibits the KLF4-centric profibrotic transcriptional program that promotes FMT (Jiang et al., 2025).

## 5 Non-coding RNAs

Non-coding RNAs (ncRNAs) are RNA molecules that do not code for proteins but play crucial regulatory roles in gene expression (Beermann et al., 2016). MicroRNAs (miRNAs) are short ncRNAs, approximately 20 nucleotides in length, that bind to mRNAs to inhibit translation or induce degradation. Long non-coding RNAs (lncRNAs) are longer ncRNAs, typically exceeding 200 nucleotides, and can regulate gene expression through various mechanisms, including chromatin remodeling and transcriptional interference (Yao R. W. et al., 2019). Circular RNAs (circRNAs) form covalently closed loops and can act as miRNA sponges, sequestering miRNAs and modulating their activity. ncRNAs contribute to PF mainly via post-transcriptional regulation and competing endogenous RNA (ceRNA) networks that is specific to cell types (Beermann et al., 2016; Yao R. W. et al., 2019; Shang et al., 2023).

### 5.1 miRNAs

**FIGURE 4:**
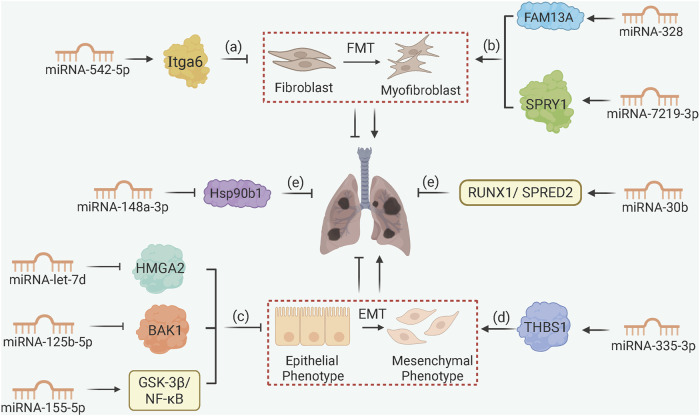
MicroRNA Networks Controlling Fibroblast and Epithelial Cell Fate. (a) miRNA-542-5p targets Itga6 to inhibit FMT in silica-induced PF; (b) macrophage-derived exosomal miRNA-328 and miRNA-7219-3p accelerate FMT by targeting FAM13A and SPRY1, respectively; (c) miRNA-let-7d, miRNA-125b-5p, and miRNA-155-5p inhibit EMT by targeting HMGA2, BAK1, and GSK-3β/NF-κB, respectively; (d) miRNA-335-3p downregulation upregulates THBS1 to drive EMT progression; (e) MSC-derived exosomal miRNA-148a-3p and miRNA-30b suppress fibrosis via Hsp90b1 and RUNX1/SPRED2 pathways.

miRNAs control the expression of over 60% of coding genes, primarily by attaching to the 3′UTR of mRNAs (Shang et al., 2023). In PF, miRNAs regulate FMT and EMT by targeting key signaling nodes (Figure 4). For example, miRNA-542-5p inhibits FMT by targeting Itga6 (integrin α6), disrupting FAK/PI3K/AKT signaling and reducing fibroblast migration in silica-induced PF (Yuan et al., 2018). The miRNA-let-7 family (e.g., miRNA-let-7d) suppresses EMT by inhibiting HMGA2 (high mobility group A2) in silica-exposed cells, while miRNA-125b-5p and miRNA-335-3p target BAK1 and THBS1, respectively, to block mesenchymal transition (Yu et al., 2019; Zhou et al., 2024; Han et al., 2024). Radiation exposure induces PF by downregulating miRNA-155-5p, thereby activating GSK-3β/NF-κB signaling and promoting EMT (Wang et al., 2021).

Exosomal miRNAs mediate intercellular crosstalk in PF as well. Macrophage-derived exosomal miRNA-328 and miRNA-7219-3p accelerate FMT by targeting FAM13A and SPRY1 in fibroblasts, respectively (Yao M. Y. et al., 2019; Niu et al., 2022). In contrast, MSCs-derived exosomal miRNA-148a-3p and miRNA-30b inhibit fibrosis by targeting Hsp90b1 (heat shock protein 90β) and RUNX1/SPRED2, respectively, to reduce collagen synthesis and inflammation (Jiang et al., 2023; Zhu et al., 2024). The miRNA-let-7 cluster (miRNA-let-7a1, miRNA-let-7f1, miRNA-let-7d), collectively known as miRNA-let-7afd, also exhibits alveolar type II epithelial cell (AT2)-specific functions, repressing histone methyltransferase EZH2 and pro-fibrotic transcription factors (BACH1, MYC) to maintain AT2 quiescence; loss of let-7 function leads to H3K27 acetylation of these genes and aberrant fibrotic cascade (Seasock et al., 2025).

### 5.2 lncRNAs

**FIGURE 5:**
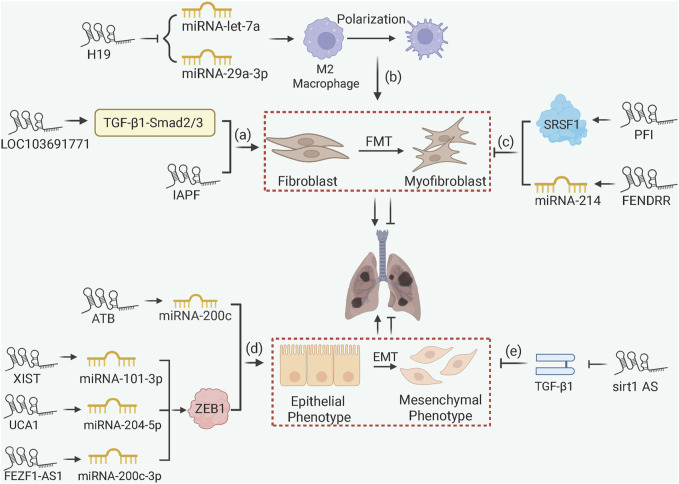
lncRNA-Mediated ceRNA and Chromatin Remodeling in PF. (a) lncRNA-LOC103691771 and lncRNA-IAPF promote FMT via TGF-β/SMAD signaling; (b) lncRNA-H19 accelerates macrophage M2 polarization and FMT via ceRNA mechanisms; (c) lncRNA-PFI and FENDRR inhibit fibrosis by binding SRSF1 to downregulate fibronectin or sponging miRNA-214 to upregulate MFN2; (d) lncRNA-ATB, lncRNA-XIST, lncRNA-UCA1, and lncRNA-FEZF1-AS1 promote EMT by sponging miRNAs to upregulate ZEB1; (e) lncRNA-sirt1-AS stabilizes SIRT1 to inhibit EMT in IPF.

LncRNAs influence PF through chromatin remodeling and ceRNA mechanisms (Figure 5). Pro-fibrotic lncRNAs such as lncRNA-LOC103691771 and lncRNA-IAPF promote FMT via TGF-β/SMAD signaling (Cai et al., 2020; Zhang J. et al., 2022), with lncRNA-H19 acting as a ceRNA to sponge miRNA-let-7a or miRNA-29a-3p thus accelerates macrophage M2 polarization in arsenic-induced PF (Xiao et al., 2021; Bu et al., 2023). Within EMT, lncRNA-ATB, lncRNA-XIST, and lncRNA-UCA1 sequester miRNA-200c, miRNA-101-3p, and miRNA-204-5p, respectively, to liberate Zinc finger E-box binding homeobox 1 (ZEB1), a crucial EMT transcription factor, thus promoting mesenchymal transition in silica-exposed models (Liu et al., 2018; Li C. et al., 2022; Yang G. et al., 2022). Similarly, in TGF-β1 cell models, lncRNA-FEZF1-AS1 promotes ZEB1 expression by sponging miRNA-200c-3p (Liu et al., 2024). It is noteworthy that not all lncRNAs promote EMT during PF progression. For example, lncRNA-sirt1-AS inhibits EMT by stabilizing sirt1 protein expression (Qian et al., 2020). Anti-fibrotic lncRNAs also include lncRNA-PFI, which directly binds splicing factor SRSF1 to inhibit pro-fibrotic splicing of fibronectin, and lncRNA-FENDRR, which sponges miRNA-214 to upregulate MFN2 (mitofusin 2) and mitigate mitochondrial dysfunction in fibroblasts (Sun et al., 2021; Liao et al., 2024).

### 5.3 circRNAs

**FIGURE 6:**
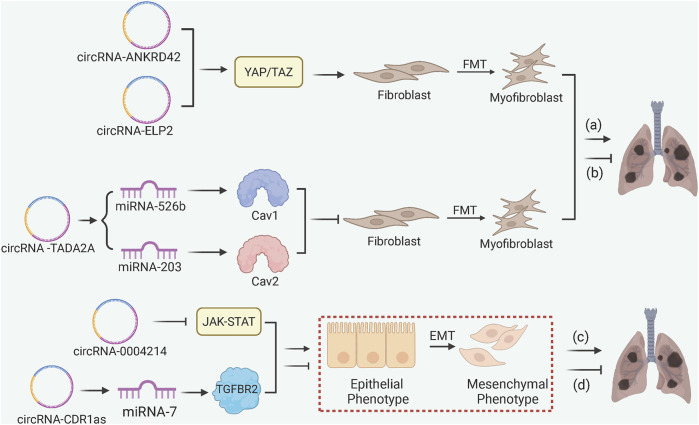
circRNA-Mediated ceRNA and Mechanical Signal Transduction in PF. (a) pro-fibrotic circRNA-ANKRD42 and ELP2 enhance FMT and ECM deposition via YAP/TAZ signaling; (b) anti-fibrotic circRNA-TADA2A inhibits FMT through miRNA-526b/Cav1 and miRNA-203/Cav2 axes; (c) circRNA-0004214 alleviates EMT by inhibiting the JAK-STAT pathway in BeSO_4_-induced fibrosis; (d) circRNA-CDR1as accelerates EMT by sponging miRNA-7 to release TGFBR2.

In general, most circRNAs appear to regulate PF through ceRNA-mediated gene sponging (Figure 6) (Yang et al., 2021; Wei et al., 2023). The anti-fibrotic circRNA-TADA2A inhibits the FMT by sequestering miRNA-526b and miRNA-203, subsequently leading to the release of Caveolin-1 (Cav1) and Cav2, respectively, to suppress the myofibroblast activation (Li et al., 2020). Both pro-fibrotic circRNA-ANKRD42 and circRNA-ELP2 promote the YAP/TAZ-mediated mechanotransduction activity, augmenting ECM deposition in response to mechanical stiffness (Xu et al., 2022; Zhang et al., 2024). circRNA-CDR1as sponges miRNA-7 to upregulate TGFBR2 and accelerate EMT in response to silica exposure, while circRNA-0004214 alleviates EMT by impeding JAK-STAT signaling in response to BeSO_4_ (Yao et al., 2018; Jin et al., 2024).

## 6 Summary and perspectives

Epigenetic regulation in PF forms an integrated cross-talk between DNA and RNA methylation, histones, and ncRNAs that result in fibroblast activation, EMT, immune cell dysregulation, and others. One feature of epigenetics in PF that is indeed the keystone is the convergence of different epigenetic mechanisms to one common set of targets. For example, lncRNA-ATB, lncRNA-XIST, lncRNA-UCA1, and lncRNA-FEZF1-AS1, where all these lncRNAs sponge different miRNAs (miRNA-200c, miRNA-101-3p, miRNA-204-5p, and miRNA-200c-3p, respectively) in order to upregulate ZEB1, a main regulator of EMT, in silica-induced PF, arsenic-induced PF and in TGF-β1-induced PF. This redundancy illustrates the strength of epigenetic in driving fibrosis. On the other side, members of the miRNA-let-7 family are prime examples of context-specific roles (i.e., they have opposite function depending on the cell type). In AT2 cells, miRNA-let-7afd keeps alveoli quiescent by repressing EZH2 and BACH1 via an acetylated H3K27. While in fibroblasts, it represses HMGA2 via miRNA-let-7d, and this reduces EMT. While those examples are explanatory, this field needs comprehensive single-cell epigenetic profiling to pinpoint therapeutic targets at cell-type specificity.

Additional heterogeneity in PF epigenetics originates from the choice of experimental models. For instance, the same gene, METTL3, drives fibrosis in silica-exposed mice due to METTL3-enhanced myofibroblast m6A methylation (Zhang et al., 2021), however it facilitates post-pneumonectomy lung regeneration over fibrosis by methylation of Foxo1 mRNA to maintain endothelial glycolysis (Ma et al., 2024). This highlights the necessity to carefully validate epigenetic mechanisms across PF subtypes, including IPF, occupational PF such as silica-related cases, as well as environmental PF such as PM2.5-induced cases.

The use of the plasticity of epigenetic pathways gives the field the opportunity to move towards precision medicine for PF, in which therapies are designed to target the dynamic relationship among the patient’s genetic predisposition, environmental insult and cell type-specific epigenetic status. The possible future technical advances, including single-cell epigenetics, spatial multi-omics and the identification of epigenetic biomarkers, should reach the final goal, that is, find interventions capable to break fibrotic cascades but preserve regenerative mechanisms, giving new hope for this life-threatening disease.
